# Transcription Factors Interplay Orchestrates the Immune-Metabolic Response of *Leishmania* Infected Macrophages

**DOI:** 10.3389/fcimb.2021.660415

**Published:** 2021-04-07

**Authors:** Haifa Bichiou, Cyrine Bouabid, Imen Rabhi, Lamia Guizani-Tabbane

**Affiliations:** ^1^ Laboratory of Medical Parasitology, Biotechnology and Biomolecules (PMBB), Institut Pasteur de Tunis, Tunis, Tunisia; ^2^ Faculty of Sciences of Tunis, Université de Tunis El Manar, Tunis, Tunisia; ^3^ Biotechnology Department, Higher Institute of Biotechnology at Sidi-Thabet (ISBST), Biotechpole Sidi-Thabet- University of Manouba, Tunis, Tunisia

**Keywords:** *Leishmania*, macrophages, transcription factor, immune response, metabolic response

## Abstract

Leishmaniasis is a group of heterogenous diseases considered as an important public health problem in several countries. This neglected disease is caused by over 20 parasite species of the protozoa belonging to the *Leishmania* genus and is spread by the bite of a female phlebotomine sandfly. Depending on the parasite specie and the immune status of the patient, leishmaniasis can present a wide spectrum of clinical manifestations. As an obligate intracellular parasite, *Leishmania* colonize phagocytic cells, mainly the macrophages that orchestrate the host immune response and determine the fate of the infection. Once inside macrophages, *Leishmania* triggers different signaling pathways that regulate the immune and metabolic response of the host cells. Various transcription factors regulate such immune-metabolic responses and the associated leishmanicidal and inflammatory reaction against the invading parasite. In this review, we will highlight the most important transcription factors involved in these responses, their interactions and their impact on the establishment and the progression of the immune response along with their effect on the physiopathology of the disease.

## Introduction

*Leishmania* spp. are protozoan parasites that affects millions of people around the world. They are the causative agent of leishmaniasis, a group of heterogeneous diseases endemic in various countries. Depending on the parasite specie, and the immune status of the patient, leishmaniasis can present a spectrum of clinical manifestations. In the mammalian host, this parasite targets the immune cells that represent the first line of defense against infections and uses macrophage as a final host making *Leishmania*-macrophages interactions central to the outcome of the disease.

Different *Leishmania* species trigger distinct immune responses and macrophage polarization, upon their infection by the parasite, highly impact the infection outcome.

In the site of infection, recruited monocytes differentiate into macrophages that show extensive plasticity, depending on converging signals delivered by the numerous factors (microbial products, cytokines (IFNγ, TNFα) and growth factors, present in the local tissue environment. Macrophages would then adopt a spectrum of distinct polarization states, among which the M1 inflammatory and the M2 anti-inflammatory cells are the extremes (Xue et al., 2014).

The « classically activated » or M1 macrophages polarization are important for the control of infections by intracellular pathogens. They are characterized by the production of proinflammatory cytokines such as TNFα, IL-1β, IL-6, IL-12, IL-18, IL-23, and Type 1 IFN, but also by the generation of reactive oxygen and nitrogen species that ensure efficient microbial killing. The « alternatively activated” M2 macrophages are elicited by type 2 cytokines (IL-4, IL-10, IL-13) and are inflammation-resolving macrophages with anti-parasitic and tissue repair functions (Murray, 2017).

These macrophage polarization phenotypes are also characterized by two distinct metabolic signatures. Indeed, in resting macrophages, glucose is metabolized to pyruvate which is transported into the mitochondria where it is oxidized into acetyl coenzyme A (acetyl-CoA) to fuel the tricarboxylic acid (TCA) cycle. The latter generates NADH, which provides electrons to the mitochondrial electron transport chain so that oxidative phosphorylation (OXPHOS) can progress and generate ATP. Upon infection, the metabolic profile switches from oxidative phosphorylation to aerobic glycolysis and the macrophages rely on glycolysis for the production of ATP (Kelly and O’Neill, 2015). Glycolysis provides the glucose-6-phosphate, an enhancer of the Pentose Phosphate Pathway (PPP) and a booster of NADPH production. This latter is then used by the NADPH oxidase for ROS and nitric oxide generation, two pathogens-killing agents. Metabolism reprogramming embraces also different other metabolism including lipid, and M1 macrophages have been shown to drive fatty acid synthesis (Zhu et al., 2015). By contrast, M2 macrophages rely on fatty acid oxidation in the mitochondria to generate acetyl-CoA, NADH, and FADH2 used to produce ATP through OXPHOS, assuring the long-term need in energy of these macrophages.

Along with this role in energy generation, cellular metabolism is emerging as an important determinant of the functional phenotype acquired by macrophages and a key regulator of macrophage immune response and inflammation (Kelly and O’Neill, 2015; Zhu et al., 2015). For instance, the role of L-arginine metabolism in regulating the microbicidal activity of macrophages through the production of nitric oxide or the generation of ornithine and polyamines is well documented and was one of the first characteristics used to define macrophage subsets (Muxel et al., 2017).

Progress has been made in defining the molecular pathways that underlie M2 versus M1 polarization and the different signaling molecules and transcription factors involved in regulating macrophage metabolic profiles, polarization and function.

Functional control of macrophages largely occurs at the transcriptional level and different transcription factors have been implicated in the polarization and activation of macrophages in response to infection. Signal transducers and activators of transcription (STATs) (STAT1), interferon regulatory factors (IRF) and nuclear factor kappa B (NF-κB) together with hypoxia-Inducible Factor (HIFα), are major players in inflammation and in the M1 polarization of macrophage during infection whereas IRF4, STAT6 and Peroxisome proliferator-activated receptor (PPAR) activated by IL-4, control M2 gene expression (Murray, 2017).

In this review we will summarize key mechanisms of *Leishmania*-macrophage interactions, focusing on the contribution of transcription factors (**Table 1**) to macrophage responses and to the physiopathology of the disease.

**Table 1:** Key transcription factors that modulate macrophages response to *Leishmania* infection.

Transcription Factors	upstream regulators	Biological Effects	References
IRFs	TLR	Macrophage polarization	(Honda et al., 2005; Negishi et al., 2005)
NF-κB	TLR/TNFα/IL-1Rc-TRAF-IKK	Immune response-Inflammation	(Reinhard et al., 2012)
HIF-1α	Hypoxia, TLR	Increases Glycolysis-Inflammatory, immune responses	(Schatz et al., 2018)
NF-AT	TLR2-PI3K-PLC-Calcineurin	Immune response	(Bhattacharjee et al., 2016)
SREBP	PI3K/Akt/mTOR	cholesterol biosynthesis and lipid homeostasis	(Mesquita et al., 2020)
NRF2	PI3K/Akt/PKR…	Response to oxidative stress	(Vivarini and Lopes, 2019)
PPAR	Oxidized fatty acids and their derivatives	Macrophage polarization	(Bouhlel et al., 2007; Díaz-Gandarilla et al., 2013)
LXR	Oxysterols	Decreased fatty acids and cholesterol synthesis	(Leopold Wager et al., 2019)

## Transcriptomic Signature of *Leishmania* Infected Macrophages

When infecting macrophages, microbes induce marked changes in gene expression programme and shape the phenotype and function of the infected cell. These changes have been investigated for different intracellular pathogens and the macrophagic transcriptomic signature identified for various bacteria, viruses and parasites. Different genes expression profiles have been compared and pathogens-mediated signaling have been shown to share a common host-transcriptional-response (Jenner and Young, 2005), which includes the group of genes that activate immune response, genes encoding inflammatory cytokines and chemokines and those stimulated by Interferon. This common host transcriptional-response is also shared by the transcriptomic profile of *Leishmania* infected macrophages (Buates and Matlashewski, 2001; Chaussabel et al., 2003; Rodriguez et al., 2004; Osorio y Fortea et al., 2009) except for the IFNγ activated genes involved in the development of the microbicidal activities and promoted by the Janus kinase signal transducer and activator of transcription (JAK/STAT) pathway.

Interferon gamma receptor ligation induces the activation of JAK1 and JAK2 tyrosine kinases, constitutively associated to the receptor. These kinases phosphorylate, on the receptor, specific tyrosine residues that serve as a docking site for STATI transcription factor which is also a target of JAKs. Once phosphorylated by JAKs, STATI form dimers and translocate to the nucleus to bind the gamma interferon activation site (GAS) elements, present on different promoters of several genes including those encoding for NOS2, the MHC class II transactivator (CIITA) and IL-12 cytokine.

*Leishmania*-infected macrophages fail to respond to IFNγ a key cytokine that empower macrophages to clear the parasite and this failure is the result of the IFNγ signaling pathway inhibition. Indeed, in both differentiated U-937 cells and human monocytes infected by *Leishmania donovani*, the tyrosine phosphorylation, Jakl and Jak2 activation and Statl phosphorylation normally induced by IFNγ, were selectively impaired (Nandan and Reiner, 1995). Similar response was observed when macrophages were infected by other *Leishmania* species. Several mechanisms have been proposed to explain the inhibition of JAK-STAT pathway during *Leishmania* infection. This IFNγ signaling pathway inhibition was suggested to be the result of a decreased expression of IFN-gamma R-alpha protein after infection (Ray et al., 2000). The co-immunoprecipitation of JAK2 with the *Leishmania* induced-tyrosine phosphatase (P’TP) SHP-1, suggests that this phosphatase may be at the origin of the IFN-gamma-inducible JAK2 unresponsiveness (Blanchette et al., 1999). However, additional mechanisms must participate to the inhibition of IFNγ signaling as *Leishmania* parasite retains its capability to inhibit STATI activation in SHP-1-deficient macrophages suggesting a SHP-1 independent mechanism (Forget et al., 2006). *Leishmania* induced STATI inactivation could be also the consequence of proteasome-mediated degradation of this transcription factor since proteasome inhibitors rescued STAT1 nuclear translocation and restored STAT1 protein level (Forget et al., 2005). It could also be the result of a defective nuclear translocation of STATI due to a compromised IFN-induced STAT1 association with the nuclear transport adaptor importin-5 as described for *L. donovani* amastigote (Matte and Descoteaux, 2010).

The suppressor of cytokine signaling (SOCS) proteins by binding to JAK or cytokine receptors and suppressing STAT phosphorylation are one of the crucial negative regulators of JAK/STAT signaling pathways. SOCS have been reported to be activated in response to different parasites (Bullen et al., 2003a; Zimmermann et al., 2006; Dalpke et al., 2008) and to play a role in regulating immune response in various infectious disease including leishmaniasis (Bullen et al., 2003b). Indeed, *Leishmania* LPG, an agonist of TLR2 (de Veer et al., 2003) and *L. donovani* parasites have been shown to induce SOCS3 and SOCS1 expression in macrophages (Bertholet et al., 2003). The early and transient expression of SOCS3 may suppress the reactive oxygen species-mediated apoptotic signaling cascade (Srivastav et al., 2014) while the stable and robust expression of SOCS1 could inhibit phosphorylation of STAT1 and induce the down-regulation the IL-12 cytokine level in infected macrophages (Chandrakar et al., 2020).

Hence, in response to *Leishmania* infection, several mechanisms, acting at various steps of the JAK-STAT pathway might potentially cooperate to coordinately shutdown JAK-STAT pathway, crucial for macrophage-T cells cross talk.

## Transcription Factors Underlying *Leishmania*-Induced Inflammatory Response

Macrophages sense protozoan parasites using different surface receptors that participate in the course of infection and have different effect on macrophage-*Leishmania* interaction. Among these, the Toll like receptors (TLRs), activated by various parasites surface molecules, play a crucial role in initiating host innate immune responses and directing adaptive immune responses against invading pathogens. TLRs localize variously as TLR1, -2, -4, -5, and 6, are expressed on the cell membrane, whereas TLR3, -7, -8, -9, are expressed in the endosome. Once activated adaptor molecules, such as MyD88 (myeloid differentiation primary-response protein 88) shared by all TLRs except for TLR3, TRIF (TIR-domain-containing adaptor protein inducing beta interferon (IFN-β), unique to TLR3 and TLR4 signaling, TIRAP (TIR-domain-containing adaptor protein) that mediates the activation of signaling downstream of TLR2 and TLR4, or TRAM (TIR-domain-containing adaptor molecule) unique to TLR4, are recruited to the receptor (Satoh and Akira, 2016).

Different TLRs such as TLR2, TLR4 (Kropf et al., 2004) and TLR9 have been involved in providing protection against *Leishmania* spp infection (Liese et al., 2007; Gurung and Kanneganti, 2015). It was therefore not surprising that mice deficient for MyD88, the common adaptor acting downstream of almost all the TLRs, were highly susceptible to *L. major* infections (de Veer et al., 2003; Debus et al., 2003; Muraille et al., 2003).

The interaction between the *Leishmania* ligands and TLRs induce the recruitment of different adaptor molecules that transduce TLRs intracellular signals and lead to the activation of various transcription factors, including NF-κB and IRFs which play a key role in defining functional phenotype of macrophages (Colonna, 2007).

Indeed, MyD88 adaptor has been shown to form a multimolecular complex consisting of IRAKI, IRAK4, TRAF6, IRF-5, and IRF-7. IRF7 is a master regulator of type I IFN genes (Honda et al., 2005) whereas IRF5 is implicated in TLR-dependent induction of pro-inflammatory cytokines, such as interleukin-6 (L-6), IL-12p40 and tumour-necrosis factor-alpha (TNFα) as confirmed by IRF5 deficient mice (Takaoka et al., 2005). IRF-5 that play an essential role in M1 macrophages polarization, compete with IRF4 for binding to MyD88 (Ferrante and Leibovich, 2012). This negative regulator of MyD88 signaling is crucial for M2 polarization and IRF4 and IRF5 competition for binding to MyD88 plays a key role in choosing whether macrophage polarization goes toward M1 or M2 phenotype (Negishi et al., 2005).

The TRIF-dependent pathway, restricted to TLR-3 and TLR-4, recruits IRF3 to induce type I IFN responses, particularly IFN-beta (Sharma et al., 2003).

IRF1, highly induced by IFNγ, directly interacts with and is activated by MyD88 upon TLR9 activation (Schmitz et al., 2007) and the TLR9-mediated IRF1 activation, as confirmed in IRF1-deficient cells, is required for optimal regulation of a specific gene subset that includes IFNβ, inducible NO synthase (iNOS) and IL-12p35 (Negishi et al., 2006).

Hence multiple TLR pathways require either IRF-1, IRF-3 or IRF-7 for type I IFN-induction. IRF-1 and IRF-7 are recruited and activated through MyD88, which simultaneously triggers IRF-5 and a parallel signaling pathway for NF-κB activation leading to secretion of proinflammatory cytokines (Colonna, 2007).

IRF-1 (Lohoff et al., 1997), IRF-2 (Lohoff et al., 2000) and IRF-8 (Giese et al., 1997) knockout mice fail to mount a protective Th1 response and are thus highly susceptible to *L. major* infection. However, a defect in IRF1 expression is also observed in *Leishmania* infected macrophages treated with IFNγ (Matte and Descoteaux, 2010). This defect may be due to the inhibition of STAT1 activation by *Leishmania* infection. Indeed, IRF1 expression is mediated by STAT1 and NF-κB transcription factors, a finding confirmed by the data showing that IFN-induced expression of IRF-1 mRNA was completely abolished in STATl deficient cells (Meraz et al., 1996). Similarly, IRF5 was shown to be required for the development of host-protective Th1 responses in response to *Leishmania donovani*. However, in IRF5-/- mice (Paun et al., 2011) and in myeloïd CD11c specific IRF5-/- mice, *Leishmania* failed to induce splenomegaly which seems to be detrimental to the host (Mai et al., 2019).

Besides IRFs, the TLR MyD88/TIRAP signaling pathways culminate in activation of the transcription factor nuclear factor-kappaB (NF-κB) *via* either classical pathway engaging the IKK-related kinases TBK1 (TRAF family member-associated NF-κB activator (TANK) binding kinase-1) downstream of TRAF6 or alternative pathways (Kawai and Akira, 2007).

NF-κB transcription factor family has five different members: NF-κB1(p50), NF-κB2 (p52) (formed from a larger precursor, respectively p105 et p102), p65 relA, c-rel and rel-B. They are all able to operate as a homo- or hetero-dimers and potentially yield to 15 different NF-κB complexes. These NF-κB dimers bind an inhibitory protein “inhibitor of κB” (IκB) and are trapped in the cytoplasm. The phosphorylation of IκB induces its ubiquitination and degradation and result in the release and the nuclear translocation of this master transcription factor that regulates hundred of genes. A large bulk of these genes are involved in both immediate and delayed expression of inflammatory mediators making NF-κB transcription factor a key regulator of proinflammatory pathway (Reinhard et al., 2012), NADPH oxidase expression and mitochondrial activity.

NF-κB pathway plays a crucial role in host protection against *Leishmania* infection and the deficiencies in different NF-κB family members in mice result in high susceptibility to *Leishmania* infection. Indeed, a defect in relB induces a multi-organ inflammation in mice, underlying the inhibitory effect of relB protein (Weih et al., 1995). Similarly, NF-κB2 deficient mice, with resistant background, develop chronic non healing lesions associated with uncontrolled parasite replication as a result of a reduced CD40-induced IL-12 production by macrophages (Speirs et al., 2002). RelA deficiency is fatal and induces the death of mice during the fetal life. However, RelA-/- chimeric mice present defects in macrophage function and show a reduced production of NO and an inability to control parasite replication and to clear infection (Mise-Omata et al., 2009). While relA is implicated in the regulation of iNOS expression, c-rel seems to regulate IL-12 production and c-Rel-deficient mice were unable to resolve *Leishmania* infection in the absence of IL-12 recombinant treatment (Grigoriadis et al., 1996; Sanjabi et al., 2000; Abu-Dayyeh et al., 2010).

In macrophages, *Leishmania* parasite seems to be unable to activate p65 NF-κB subunit. Indeed, *Leishmania Mexicana* promastigotes induce the cleavage of p65 relA subunit and generate a smaller p35 protein whereas the amastigote form fully degrades relA (Abu-Dayyeh et al., 2010). *Leishmania amazoniensis* induces the p50/p50 repressor complex which leads to a reduced activation of nitric oxide synthase (iNOS) (Calegari-Silva et al., 2015). From his side, *Leishmania major* down-regulates relA containing complexes but induces a c-rel containing complexes that retain the ability to strengthen the innate immune response of the host through the regulation of proinflammatory cytokines expression (Guizani-Tabbane et al., 2004).

NF-κB regulates the expression of myriads of cytokines and chemokines including IL-1β that play crucial role in cellular defense. This cytokine is produced as pro-IL-1β which is processed and released by the NLRP3 inflammasome activated caspase1. Different species of *Leishmania* have been reported to induce NLRP3 inflammasome, that have been shown to play a key role in restricting *Leishmania* replication in macrophages (Lefèvre et al., 2013; Lima-Junior et al., 2013) although detrimental effects have been reported for certain *Leishmania* strains such *L. major* Seidman strain or *L. braziliensis* for which disease severity is associated to IL-1β production (Charmoy et al., 2016; Santos et al., 2018). It is therefore consistent that several *Leishmania* species actively inhibit inflammasome formation and have developed different strategies to inhibit NF-κB-dependent inflammatory response. This was achieved in part by targeting the cytosolic inducible protein A20, also known as TNFα-induced protein 3 that de-ubiquitinates TRAF6 to inhibit IκB degradation and hence NF-κB activation reducing the extend of NLRP3 inflammasome activation (Boone et al., 2004; Gupta et al., 2017). Recent study highlights a key role for epigenetic modifications at the promoter of NF-κB-regulated genes that dampen inflammasome activation (Lecoeur et al., 2020). Indeed, *Leishmania amazoniensis* amastigotes effector molecules, target histone H3 post-translational acetylation, to reduce expression of positive regulators of the NF-κB pathway and upregulate transcripts of anti-inflammatory molecules and known inhibitors of NF-κB activation, leading to the inhibition of NF-κB-NLRP3 axis.

## Transcription Factor HIF-1α and *Leishmania*-Induced Metabolic Reprograming

Since a decade, the importance of metabolic reprogramming in macrophages fighting microbes has emerged (Chauhan and Saha, 2018; Wang et al., 2019). Indeed, low oxygen levels have been described in the areas of myeloid cell activity and site of inflammation. Studies extending back almost a century have demonstrated that macrophages in these areas are highly dependent on anaerobic glycolysis for the production of ATP. This glycolysis surge and reduced glucose mitochondrial oxidation have been shown to depend on HIF-α transcription factor, which plays a key role in assisting macrophage to adapt to hypoxic conditions and achieve their microbicidal function (Marín-Hernández et al., 2009).

Under hypoxia, the inactivation of the oxygen-sensitive enzymes prolylhydroxylases (PHDs) allows HIFα (one of the HIF-α isoforms: HIF-1α or HIF-2α) to escape proteosomal degradation. HIF-α accumulates and translocates to the nucleus where it forms a heterodimer with the constitutively expressed HIF-1ß subunit. The HIFα/β heterodimer binds to the hypoxia response elements (HREs) and drive then the expression of different genes (Ke and Costa, 2006). HIF-1α is a direct transcriptional activator of glucose transporters genes involved in glucose uptake as well as keys glycolytic and gluconeogenesis enzymes. It also decreases mitochondrial respiration and thus oxygen consumption by upregulating the expression of pyruvate dehydrogenase kinase (PDK) (Bishop and Ratcliffe, 2014) that limit the formation of acetyl-CoA. Glycolysis even less efficient than oxidative phosphorylation for ATP generation can be promptly ramped up to sustain ATP production and meet the rapid energy requirement of macrophages (Nagao et al., 2019).

Similarly to other site of infection, *Leishmania* infected skin has been demonstrated to be hypoxic. This low oxygen level has been described for *L. amazonensis* and *L. major* infected tissue (Araujo et al., 2013) and resolution of the wound was reported to correlate with reoxygenation (Mahnke et al., 2014).

HIF-lα expression can also occur in an oxygen-independent manner in myeloid cells fighting pathogens, driving metabolic reprogramming to modulate oxygen consumption and modulating innate immunity (Jantsch and Schödel, 2015; Devraj et al., 2017; Stothers et al., 2018; Knight and Stanley, 2019). *Leishmania* parasite also induces HIF-1α activation (Charpentier et al., 2016; Schatz et al., 2018). Indeed, the HIF-lα has been detected in the cutaneous lesions of Balb/c mice (Arrais-Silva et al., 2005) and in the nucleus of *L. amazonensis* infected mouse peritoneal macrophages and human MDM (Degrossoli et al., 2007). HIF-1α also accumulates in *L. donovani* infected macrophagic cell lines (Singh et al., 2012) under normoxic conditions. In our hand, *L. major* induces the accumulation of HIF-la in BMDM cells as assessed by confocal microscopy (unpublished data). A more recent study has however shown that *L. major* infection induces HIF-lα accumulation in the macrophage-rich cutaneous *Leishmania* lesions of mice and humans, but needs, *in vitro*, additional stimulation such IFNγ (Schatz et al., 2016).

Moreover, different HIF1α target genes including glucose transporters assuring glucose uptake, glycolytic enzymes and pyruvate dehydrogenase kinase 1 (PDK1) leading to inactivation of the TCA cycle are induced in *Leishmania* infected macrophages (Rabhi et al., 2012; Moreira et al., 2015). These allow the metabolic reprogramming of infected macrophages toward aerobic glycolysis and hence increased glucose uptake, activation of the pentose phosphate pathway (PPP) that fuels NADPH to activate NADPH oxidase for ROS generation, enhanced lactate production and decreased mitochondrial oxygen consumption.

Activation of HIF1α has contrasting effects on the pathogen survival. Unlike for bacterial and for some viral infections (Devraj et al., 2017), HIF-lα activation during several parasitic infections could promote the intracellular survival of parasites. Indeed, HIF-la stabilization is required for the survival of *Toxoplasma gondii* (Wiley et al., 2010); Similarly, *Theileria annulata* induces hif1-α transcription and drive host cell toward aerobic glycolysis limiting the production of H202 and hence protecting the parasites in infected macrophages (Metheni et al., 2014).

The impact of HIF1α on *Leishmania* parasite survival has been addressed in several studies and has yielded contrasting results. Indeed, Singh et al., using macrophagic cell lines, show that knockdown of HIF-lα inhibited intracellular growth whereas over expression of this TF promotes intracellular growth of *L. donovani* parasites (Singh et al., 2012). However, different other studies show that HIF-1 alpha is a host protective factor against infection by *Leishmania* parasites. Indeed, silencing of HIF-lα in macrophages, using RNA interference, inhibited LPS/IFNy-induced NO release in response to *L. major* and abolished the leishmanicidal activity of macrophages showing that HIF-lα expression by myeloid cells contributes to the control of *L. major* parasites *in vitro* and *in vivo* (Schatz et al., 2016). Finally, the viability of *L. donovani* is enhanced in myeloïd-restricted HIF-1-/- mice (mHIF-1-/-) and in macrophages from mHIF-1-/-. This increased intracellular growth both *in vitro* and *in vivo*, was the result of enhanced lipid synthesis induced by a defect in the expression of BNIP3 that allows the activation of the mTOR/SREBPlc axis (Mesquita et al., 2020).

Thus, besides carbohydrates metabolism regulation, HIF1α, by regulating mTOR signaling pathway, controls lipid metabolism.

HIF-1 is also linked to inflammatory response and microbicidal activities of myeloid cells. Indeed, the relationship between the hypoxic and inflammatory responses is tightly controlled and cross talk between HIF1α and NF-κB, the two main molecular players involved in hypoxia and innate immunity, has been demonstrated (Palazon et al., 2014; D’Ignazio et al., 2016). Different studies have underlined the importance of HIF-1 in regulating inflammatory cytokines. Mice with HIF-1α myeloid-specific deletion showed impaired inflammatory responses, underlining the importance of HIF1α and the high glycolytic rates and energy generation for basic myeloid cell activities (Cramer et al., 2003). Both HIFs are able to regulate a number of pro-inflammatory cytokines and chemokines directly, further contributing to the inflammation response (D’Ignazio et al., 2016) and NF-κB is critical transcriptional activator of HIF1α (Rius et al., 2008; van Uden et al., 2008).

Hypoxic response is also tightly connected to innate immune response and HIF1 and NF-κB synergistically respond against pathogens. Indeed, hypoxia and bacterial infections increased NF-kB activity in phagocytes, leading to the increase in HIF-1a mRNA transcription (Rius et al., 2008). Moreover, bacteria-infected macrophages are characterized by a defective HIF-1α expression following ablation of Iκκb, an essential regulator of NF-κB activity (Rius et al., 2008).

**Figure 1:**
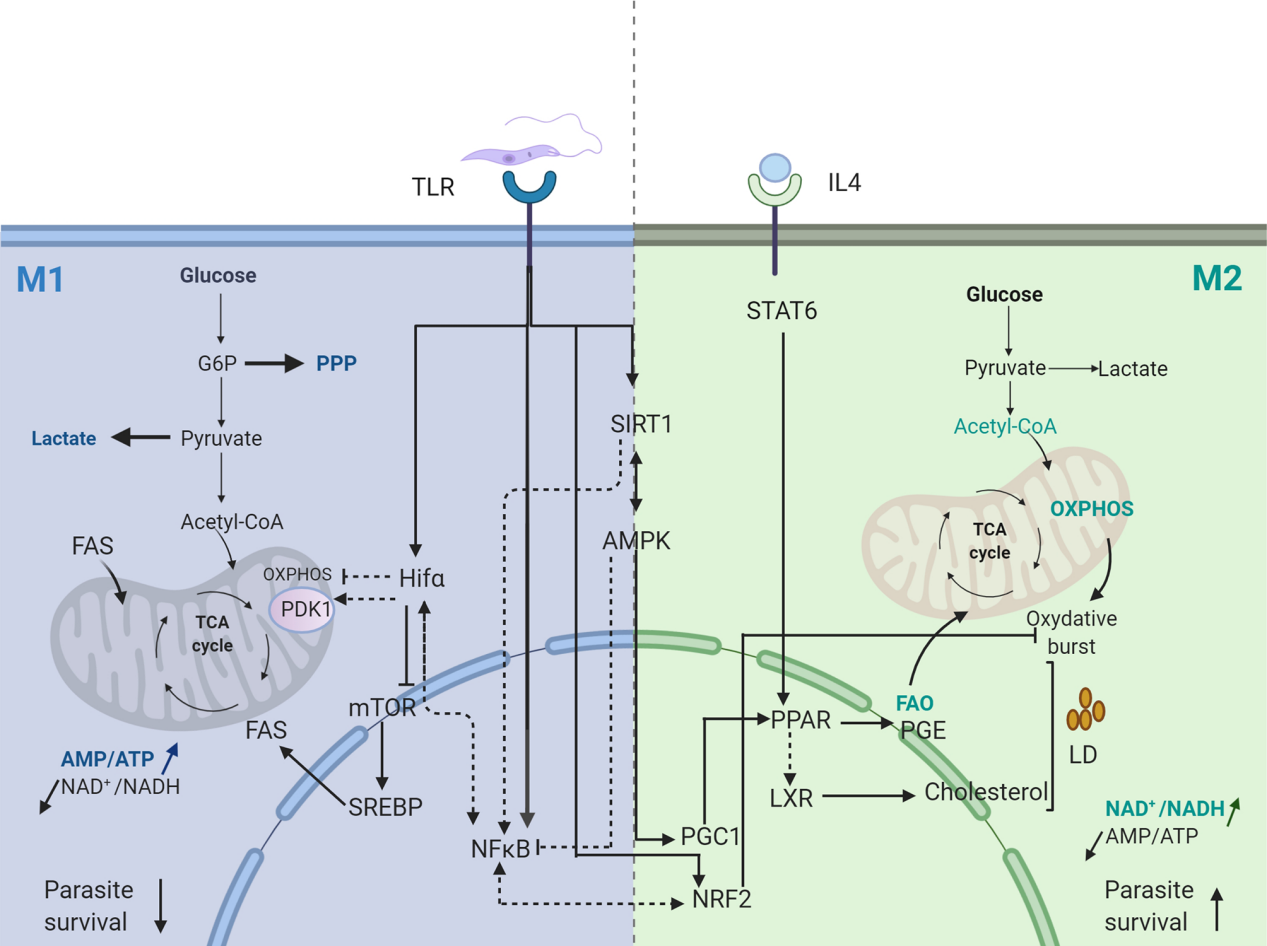
Transcriptional signature of *Leishmania* infected macrophage. Dotted arrows represent pathways not validated in *Leishmania* infected macrophages. Straight arrows represent pathways validated in *Leishmania* infected macrophages. FAS, Fatty Acid Synthesis; FAO, Fatty Acid Oxydation; PGE, Prostaglandine E; PPP, Pentose Phosphate Pathway; OXPHOS, Oxidative Phosphorylation. Created with BioRender.com.

Hence*, Leishmania* infection, by inducing NF-κB and HIF1 transcription factor, provides a bridge for regulating immune response and ignite inflammation by glucose fueling (**Figure 1**).

## Interplay Between Transcription Factors and Energy Sensors in *Leishmania*-Infected Macrophages

One target gene of NFκB is SIRT1 whose promoter sequence contains several putative NF-kB binding sites (Voelter-Mahlknecht and Mahlknecht, 2006; Mahlknecht and Voelter-Mahlknecht, 2009). Sirt1 belong to the Sirtuins family of nicotinamide nucleotide dinucleotide NAD(+)-dependent protein deacetylases that contain seven enzymatic activities in mammals (SIRT1–SIRT7) with different subcellular localisation that function to suppress gene transcription by epigenetic mechanisms. A second energy sensor, the adenosine 5’-monophosphate (AMP)-activated protein kinase (AMPK) is a key player in preserving cellular ATP.

During the early pro-inflammatory phase relying on glycolysis, decreases in ATP production and later on in NADH formation, increase the ratios of AMP/ATP, and NAD^+^/NADH. The increased AMP/ATP ratio induces AMPK while the enhanced cellular NAD+ activates SIRT proteins. These two pathways, can act in concert to reinforce one another in order to ensure the metabolic homeostasis of the cell. Indeed, On one hand SIRT1 protein has been described as an upstream activator of the AMPK through LKB1 (the kinase that mediates the phosphorylation of AMPK) deacetylation (Lan et al., 2008) and on the other hand has been considered as a downstream target of AMPK, becoming activated by the AMPK-induced increased levels of NAD+ (Cantó et al., 2010). Moreover, SIRT1 and AMPK proteins share several downstream targets such as PGC-1α (Rodgers et al., 2005) and PPARα (Purushotham et al., 2009).

SirT1 directly interacts with and deacetylates NFκB p65 (Yeung et al., 2004) inhibiting its transactivation capacity and suppressing the transcription of NF-κB dependent genes expression (Natoli, 2009). This antagonistic cross talk between NF-κB signaling pathway and SIRT1 pathways, bridge energy metabolism with innate immunity and have crucial role in the termination of NF-κB driven inflammatory response (Kauppinen et al., 2013; Vachharajani et al., 2016).

SIRT1 also deacetylates another key player in the regulation of energy metabolism/promoting mitochondrial biogenesis, the peroxisome proliferator activated receptor γ (PPARγ) coactivator 1 (PGC1) α, upregulating its activity which is further enhanced by AMK-mediated phosphorylation (Cantó et al., 2010). PGC-1α promotes the metabolic switch toward M2 profile. Indeed, the overexpression of PGC-1 reduces the transcriptional activity of NF-κB and suppressed the expression of proinflammatory cytokines and free fatty acids (Eisele et al., 2013). PGC-1α cooperates with the peroxisome proliferator-activated receptor alpha (PPARα) to control the transcription of genes encoding mitochondrial fatty acid oxidation enzymes shifting the fuel usage from glucose to fatty acids (Vega et al., 2000). Moreover, PGC1α is also a coactivator of a wide variety of transcription factors including Forkhead box-containing protein type O 1 (FOXO1), nuclear respiratory factors (NRF)1 and NRF2, many of which are involved in the transcriptional control of mitochondrial proteins and thus in mitochondrial biogenesis (Feige and Auwerx, 2007).

Hence, these energy sensors have emerged as key regulators of inflammation and metabolic switches, playing a crucial role in macrophage metabolic reprogramming toward M2 macrophages profile during the progression of inflammatory response.

These energy sensors have been shown to play an important role in the polarization of parasite infected macrophages. Indeed, the intracellular growth of *Trypanosoma cruzi* was recently shown to rely predominantly on energy production, nucleotide metabolism and fatty acid oxidation of the host and silencing of AMPK catalytic or regulatory subunits, favors intracellular *T. cruzi* growth (Caradonna et al., 2013). Similarly, activation of the host AMPK signaling inhibits the intracellular growth of *Plasmodium falciparum* parasite (Ruivo et al., 2016). The initial transient aerobic glycolytic phase induced by *Leishmania* parasites has been shown to be followed by a metabolic shift towards mitochondrial metabolism, allowing the metabolic recovery of the host cell (Moreira et al., 2015). This metabolic switch to oxidative phosphorylation and β-oxidation, is associated with an increase in PGC-1α levels and a significant increase in the AMP/ATP ratio. This metabolic reprogramming requires the activation of AMPK downstream to SIRT1 and LKB1 and ultimately contributes to parasite survival *in vitro* and *in vivo* (Moreira et al., 2015). Hence targeting the SIRT-AMPK axis may explain how the *Leishmania* parasite subverts the host metabolism to insure its persistence and replication (**Figure 1**).

## Microenvironment Impact on Activity of PGC1 Co-Activator

Microenvironment play a role, *in vivo*, in the metabolic reprograming of *Leishmania* infected macrophages and their shift from M1to M2. Indeed, the significantly raised plasma levels of IL-4/IL-13 and IL-10 in VL patients suggested a microenvironment conducive for alternative activation of monocytes/macrophages (Roy et al., 2015). Indeed, both IL-4 and IL-13 cytokines induced the expression of the two metabolic regulators, Pparγ, Pparδ and of the coactivator protein PGC-1β, through the activation of STAT6.

The Peroxisome proliferator-activated receptors (PPARs) but also the Liver X Receptors (LXRs) nuclear receptors are two transcription factors families implicated in the regulation of macrophage lipids metabolism. Both ligand-dependent transcription factors heterodimerize with the retinoid X receptor (RXR).

The peroxisome proliferator-activated receptor-γ (PPARγ) is a master regulator that assures fatty acid homeostasis. It binds mono- and polyunsaturated fatty acids and derivatives such as eicosanoids to regulate the transcription of various genes that control lipid metabolism and fine-tune immune function. Indeed, a peroxisome proliferator response element (PRE) have been identified in all the genes that encode the rate limiting steps in the transport, synthesis, storage, mobilization, activation and oxidation of fatty acids but also in the promoter region of arginase I (Odegaard et al., 2007) and in the promoters region of phospholipase A2 (cPLA2) and cyclooxygenase-2 (COX-2), two enzymes regulating the prostaglandin production that have been often associated with anti-inflammatory activities. Phospholipase A2 (cPLA2) and cyclooxygenase-2 (COX-2) have been also reported to be activated upon TLR2 stimulation; whereas the increased macrophage cPLA2 activity induced by *Leishmania* infection is regulated at the post transcriptional level, *Leishmania*-driven COX2 induction was regulated by calcineurin and calcineurin-dependent NFAT transcription factor (Bhattacharjee et al., 2016). PPARγ inhibits the induction of a broad subset of inflammatory genes, suggesting that transrepression might be a primary mechanism responsible for the anti-inflammatory action of PPARγ.

PPARγ deficient macrophages show reduced rates of β-oxidation of fatty acids and blunted mitochondrial biogenic response and are hence unable to induce oxidative phosphorylation, showing that PPARy plays a crucial role in the differentiation of circulating monocytes toward anti-inflammatory M2 macrophages, both in humans and mice (Bouhlel et al., 2007; Odegaard et al., 2007). Similarly, the overexpression of the PPARγ transcriptional coactivators PGC-1β primes macrophages for alternative activation and prevents an M1 inflammatory response following stimulation with LPS and IFNγ (Vats et al., 2006). Indeed, the two PPAR subtypes expressed in macrophages have been implicated in the negative regulation of inflammatory responses as they inhibit pro-inflammatory gene expression including IL1β, IL6, TNFα, IL12 and iNOS (Delerive et al., 2001; Kostadinova et al., 2005).

PPARγ also activates a cholesterol efflux by inducing the Liver X receptor-alpha (LXR) expression either directly or through the induction of CYP27, which is capable of generating 27- hydroxycholesterol, an endogenous LXR activator.

Endogenous agonists binding to LXR induces conformational changes that trigger co-repressor release and co-activators recruitment, leading to transcriptional regulation of the target genes. These include those encoding the sterol regulatory element binding protein 1 (SREBPlc), the other master transcriptional regulator of fatty acid synthesis (Repa et al., 2000) but also the ATP binding cassette transporters ABCAl and ABCG1 as well as the gene encoding apolipoprotein E (APOE) (Repa et al., 2002), the cholesterol transporters that promote cholesterol efflux. LXR activation also leads to an increase in the levels of long chain polyunsaturated fatty acids (PUFAs) and triglyceride synthesis.

The Liver X Receptors, (LXRs) also play a crucial role in the macrophage anti inflammatory response promoting alternative (M2) macrophage activation. Two LXR subtypes, LXRα and LXRβ are expressed in hematopoietic cells with LXRα being restricted to the myeloid lineage. The deficient regulation of both isoforms of LXRs activity can lead to chronic inflammatory conditions. Indeed, several LXR regulated genes are targets of NF-κB and LXR agonist treatment can limit the transcriptional up-regulation of inflammatory genes such as tumor necrosis factor alpha (TNFα), cyclooxygenase 2 (COX2), inducible nitric oxide synthase (NOS2) and matrix metalloprotease 9 (MMP9) (Castrillo et al., 2003; Joseph et al., 2003).

Several mechanisms, have been proposed to explain LXR capacity to inhibit inflammation including the trans-repression of NF-κB (Ghisletti et al., 2009) and regulation of genes encoding enzymes involved in the synthesis of long chain PUFAS with known anti-inflammatory activity (Repa et al., 2000). Moreover, together with fatty acids, LXR regulates expression of LPCAT3 an enzyme that controls Fatty acyl-chain remodeling in phospholipid (Hashidate-Yoshida et al., 2015). These changes in phospholipid composition can reduce inflammation both by modulating kinase activation through changes in membrane composition and by affecting substrate availability for inflammatory mediator production (Rong et al., 2013).

Nuclear receptors (NRs) are emerging as key players in infectious diseases and targeting NRs as a novel host-directed treatment approach to infectious diseases appears to be a valid solution (Leopold Wager et al., 2019).

LXR deficiency in mice leads to reduced control of the intracellular *Mycobacterium tuberculosis* growth (Korf et al., 2009) and increased susceptibility to *Listeria monocytogenes* (Joseph et al., 2004). By contrast, mice deficient for LXR and challenged with the visceralizing species *Leishmania chagasi/infantum* had significantly decreased parasite burdens compared to C57Bl6 wild-type mice and enhanced NO production showing enhanced resistance of LXR-deficient mice to *Leishmania* infection (Ghisletti et al., 2009).

Regardless of PPARs subtypes induced by *Trypanosoma cruzi* (i.e., PPARy in murine peritoneal macrophage and PPARα in BMdM and Raw 264.7 macrophagic cell line), PPAR is involved in the *in vitro* M2 polarization of macrophages isolated from *T. cruzi*-infected mice and have a protective role in *T. cruzi* infection (Penas et al., 2015; Koo et al., 2018). In *Leishmania* infection the rôle of PPAR is controversial. PPAR agonists treatment of *L. mexicana* infected macrophages induced M1 polarization through IL-10 downregulation, cPLA2-COX-2 prostaglandins pathway deactivation and increased ROS production that control parasite burden in murine macrophages (Díaz-Gandarilla et al., 2013). By contrast, PPARy is activated by *L. donovani* both *in vivo* and *in vitro* and have been shown to promote the parasite survival, whereas the inhibition of PPAR-y facilitates *Leishmania* clearance in C57B16 peritoneal macrophages (Chan et al., 2012), suggesting that *Leishmania* parasites harness PPARγ to sustain the M2 phenotype and increase dissemination of the infection. PPARγ knockout Balb/c mice had significantly less footpad swelling 5-7 weeks after injection of *L. major* promastigotes than wild type mice, suggesting that absence of PPARγ delay disease progression in Balb/c mice (Odegaard et al., 2007). In lipid loaded *Leishmania*-infected BMDM, PPAR ligands promote *L. major* amastigote growth in an arginase-dependent manner (Gallardo-Soler et al., 2008).

Like other trypanosomatid parasites, *Leishmania* depend on their hosts for several nutrients necessary for their replication and survival. While carbohydrates have been proposed as the primary source of carbon for various parasites, including *Leishmania* (Naderer et al., 2010) various other sources have been suggested. Lipids would be a potential source especially since the infection induces the production of intracellular lipid droplets which rapidly localized in close association with the parasites (Rabhi et al., 2012; Rabhi et al., 2016).

Hence, like different other pathogens *Leishmania* parasite target metabolic pathway to scavenge nutrients to ensure its survival and to subvert the host immune response feeding two birds with one scone.

## NRF2 Antioxidant Pathway

The metabolic reprogramming toward aerobic glycolysis attenuates the activities of the respiratory chain, allowing reactive-oxygen species (ROS) production. The respiratory burst or ROS, generated by the NADPH oxydase and the mitochondrial electron transport chain after the phagocytosis of pathogens, has been recognized since decades as an essential mechanism of microbial killing. To avoid detrimental side effects and oxidative damage, host cells produce antioxidants enzymes in order to deplete ROS and RNS. The transcription of phase II defense gene including detoxifying enzymes, such as genes involved in the regeneration of glutathione (GSH), genes encoding proteins involved in sequestration of iron ions (ferritine, heme oxygenase (HO-1), genes encoding proteins responsible for the production of NADPH and anti-oxidant genes (e.g., SODs, GPx, GSH reductase), is orchestrated by the master transcription (nuclear factor (erythroid-derived 2)-like 2 (NRF2) (Suzuki and Yamamoto, 2015).

Upon cell activation, NRF2 oxidative sensor is released from its inhibitor Kelch-like ECH-associated protein-1 (Keap-1), phosphorylated by different kinases including the MAPK, phosphatidylinositol-3 Kinase (Kang et al., 2002) and double-stranded RNA-dependent protein kinase R (PKR) (Vivarini et al., 2017) and translocated to the nucleus where it binds, once associated with small Maf proteins, to the antioxidant response elements (AREs) present in the promoter region of target genes (Jung and Kwak, 2010; Ding et al., 2019; Ingram et al., 2019). This master transcription factor orchestrates the regulation of about 200 genes, working in a network to regulate various functions.

NRF2 is a key player regulating inflammation; Indeed, Nrf2 knock-out mice shows increased expression of cytokines, chemokines, and adhesion molecules and sustains the development of different inflammatory disorders (Thimmulappa et al., 2006; Kim et al., 2010). Cross talk between NRF2 and NF-κB is essential to resolve inflammatory response of the cell. Indeed, NFκB can directly regulate the induction of Nrf2 transcription, but can also by interacting with Keap1 suppress the Nrf2-ARE pathway, resulting in the repression of various genes (Wardyn et al., 2015; Bellezza et al., 2018). This inhibitory effect on macrophage inflammatory response may be also indirect, induced by NRF2-activated anti-oxidant genes. Among the antioxidative stress genes whose expression is regulated by NRF2, the Heme oxygenase 1 (HO-1) has received considerable attention. HO-1 is the rate limiting enzyme that catalysis the degradation of heme into carbon monoxide (CO) and iron and biliverdin to bilirubin. NRF2-mediated HO-1 expression, inhibited NF-κB signaling preventing the expression of inflammasome components such IL-1β and nlrp3 induced by LPS (Vitali et al., 2020). Similarly to NRF2 knockout mouse, HO-1 knockout mouse displayed chronic inflammatory disorders, are highly vulnerable to experimental sepsis induced by the classical pro-inflammatory mediator endotoxin (Wiesel et al., 2000). Human cases of genetic HO-1 deficiency are similar to those observed in HO-1 knockout mice (Kawashima et al., 2002) confirming the anti-inflammatory role of HO-1 (Paine et al., 2010). Moreover, Nrf2 expression but also HO-1 induction have been identified as a key mediator of polarization to a novel macrophage phenotype (Mox), that develops in response to oxidative tissue damage (Kadl et al., 2010). This M2 phenotype has redox and antioxidant potential and induces the expression of the anti-inflammatory and antiapoptotic Cox2, IL1β, HO-1, VEGF, and Nrf2.

Thus, the complex network of protective mechanisms against the oxidative burst orchestrated by NRF2 and the NRF2-HO-1 axis implicated in the resolution of inflammation, modulates the function and phenotype of macrophages driving macrophages polarization towards M2 phenotype (Naito et al., 2014).

The effect of NRF2 on mitochondria biogenesis may also play a role during infection. Indeed, four AREs were identified in the NRF1 (nuclear respiratory factors 1) gene promoter and were capable of Nrf2 binding (Piantadosi et al., 2008). NRF1 through TFAM (transcription factor A mitochondrial), is involved in the mitochondrial DNA replication. Moreover, crosstalk between PGC1a and NRF2 has been also recently reported highlighting a link between cellular redox and mitochondrial homeostasis.

The NRF2 pathway orchestrates the expression of anti-oxidant and cytoprotective genes. Its activity is induced upon exposure to oxidative stress to protect cell from environmental insults and to adapt to endogenous stressors.

However, it is not clear to what extend oxidative stress is crucial for pathogens clearance as several microorganisms seem to be able to survive and even thrive in an oxidative environment (Paiva and Bozza, 2014). Indeed, different bacteria and parasites can thrive in oxidative environments while antioxidants promote their clearance. *Trypanosoma cruzi* infected mice treated with several Nrf2 inducers, including cobalt protoporphyrin (CoPP), sulforaphane, N-acetyl-cysteine (NAC), or pterostilbene, had a decreased parasite burden. The inhibition of Nrf2 activity with SnPP or the promotion of oxidative stress with paraquat or H202 treatment increased *T. cruzi* parasitism, suggesting that oxidative stress fuels *Trypanosoma cruzi* infection in mice (Paiva et al., 2012). During the malaria-induced inflammatory processes, the treatment of macrophages with NRF2 inducers enhances the CD36 expression and CD36-mediated Plasmodium phagocytosis and thus the control of severe malaria through parasite clearance (Olagnier et al., 2011). Nrf2 participates in infections by other protozoan microorganisms and different study show that various parasites such *Toxoplasma gondii*, activates Nrf2 signaling pathway that is required for parasites replication (Pang et al., 2019).

The potential role of NRF2 signaling in *Leishmania* infection outcomes has been recently reviewed (Vivarini and Lopes, 2019). NRF2 antioxidant pathway is activated in response to different *Leishmania* species even if differences are observed between species. Indeed, *Leishmania amazoniensis* but also *Leishmania brasiliensis*, induce *in vitro*, NRF2 expression, *via* protein kinase R signaling (Barreto-de-Souza et al., 2015; Vivarini et al., 2017), a result confirmed in skin biopsies from *Leishmania* infected patients. Treatment of *L. major* infected BALB/c mice with NAC, a Nrf2 inducer, reduces the parasitism in their footpads (Rocha-Vieira et al., 2003), whereas depletion of glutathione, increases the burden in the footpads of C57BL/6 mice infected with *L. major* (Cruz et al., 2008). However, for *L. amazoniensis*, the Nrf2 knockdown that promotes oxidative stress, impairs parasite survival in macrophages (Vivarini et al., 2017).

Whereas NRF2-induced anti-oxidant genes, HO-1, is required for protection against *Toxoplasma gondii*  (Araujo et al., 2013), antioxidative enzymes such SOD1 and HO-1, favor the establishment of *Leishmania* infection. Indeed, *L. pifanoi* amastigotes and *L. chagasi* infection induce in macrophages an increased expression of HO-1 that promotes *Leishmania* persistence (Pham et al., 2005; Luz et al., 2012). Moreover, in mouse peritoneal and human macrophages lineages, infection by *L. amazonensis* leads to the increase of SOD1 expression in a PKR/Nrf2-dependent manner (Vivarini et al., 2017). SOD1 reduces the *Leishmania* oxidative stress and may promote the parasite survival and affect the outcome of the infection (Khouri et al., 2009; Khouri et al., 2010).

## Conclusion and Perspectives

In recent years much progress has been made in immunometabolism and there is a growing understanding of how metabolic pathways are regulated to support or direct functional changes in immune cells, controlling immunity and inflammation. Metabolic signature of immune cells including macrophages is now known to change depending on stages of development, activation state and pathological conditions. A better understanding of the metabolic mechanisms and metabolic needs of immune cells, as well as the interactions that underlie the complex metabolic and immune networks, is essential to develop promising drugs with effective metabolic interventions that enhance immune cell response.

In the context of anticancer immunotherapy, drugs targeting cancer metabolism might synergistically enhance immunotherapy *via* metabolic reprogramming of the tumor microenvironment. Different combinations of metabolic agents and immunotherapies are already in clinical trials (Li et al., 2019). Deciphering the intricate interaction existent between metabolic reprogramming and immune response has also promising therapeutic application for inflammatory and autoimmune diseases. Different small molecules targeting metabolic processes in immune cells as a strategy to limit inflammation and change the outcome of inflammatory and autoimmune diseases are currently in use clinically (Pålsson-McDermott and O’Neill, 2020). Hence, the majority of available or under development anti-rheumatic drugs, target various metabolites in immune cells belonging to purine, pyrimidine or GSH metabolism (Piranavan et al., 2020). The host directed therapies includes different promising treatment strategies targeting macrophages’ polarization to the M2 phenotype that have been found associated with atherosclerosis regression and different compound are being tested to develop more efficient antiatherosclerosis therapy (Bi et al., 2019). Infectious diseases are more complex pathologies given the additional interactions that take place between the host cell and the pathogen. Host directed therapy (HDT) targeting metabolic pathway could represent new therapeutic strategies to fight infectious diseases in the face of emergence of antimicrobial resistance or compromised host immunity. Indeed, recent studies suggest that foamy macrophages, the major contributors to Tuberculosis pathogenesis, and lipid metabolism are promising targets for HDT against Tuberculosis (Shim et al., 2020). Macrophage polarization is also an important tool in the physiopathology of leishmaniasis (Tomiotto-Pellissier et al., 2018) and targeting metabolic pathway, once a better understanding of the balance required to develop a protective response is gained, might potentially change the outcome of the infection.

Targeting transcription factors could be another promising therapeutic strategy. Indeed, deregulation of transcription factors is a common feature to the majority of human cancers and is involved in a large number of human diseases (Papavassiliou and Papavassiliou, 2016). A better understanding of the pathologies, of the mechanisms of transcriptional regulation and their precise function as well as the development of new technologies (Hagenbuchner and Ausserlechner, 2016; Hecker and Wagner, 2017; Becskei, 2020), has allowed the development of novel therapeutic strategies that target transcription factors. Different small molecules that interact in various way with transcription factors to modulate their expression, degradation, interaction with DNA or other proteins, are actually used or evaluated for cancer treatment (Lambert et al., 2018). Agents targeting transcription factors for the treatment of chronic kidney disease are under clinical trial (Hishikawa et al., 2018). Transcription factors induced in response to *Leishmania* infection play crucial role in regulating immune and metabolic responses and hence macrophage polarization. Almost all the transcription factors involved in the macrophage immune and metabolic response to *Leishmania* infection have been described as therapeutic target in different diseases including infectious ones. These includes HIF that couple immunity with metabolism (Bhandari and Nizet, 2014; Santos and Andrade, 2017), nuclear receptors in metabolic, inflammatory and infectious diseases (Rudraiah et al., 2016; Wang et al., 2017; Mirza et al., 2019) and NRF2 in cancer (Lu et al., 2016). Despite the challenges that remain to be overcome (delivery methods, compensatory and pleiotropic effects), targeting transcription factors could represent new therapeutic strategies to reprogram the macrophage response in order to amplify the microbicidal activity that should allow the fight against infectious diseases while preventing an exacerbated immune response leading to a worsening of the lesions.

## Author Contributions

All authors contributed to the article and approved the submitted version.

## Conflict of Interest

The authors declare that the research was conducted in the absence of any commercial or financial relationships that could be construed as a potential conflict of interest.
